# Identification and Structural Characterization of the N-terminal Amyloid Core of Orb2 isoform A

**DOI:** 10.1038/srep38265

**Published:** 2016-12-06

**Authors:** Silvia A. Cervantes, Thalia H. Bajakian, Maria A. Soria, Alexander S. Falk, Rachel J. Service, Ralf Langen, Ansgar B. Siemer

**Affiliations:** 1 Department of Biochemistry & Molecular Medicine and the Zilkha Neurogenetic Institute, Keck School of Medicine of USC, 1501 San Pablo St, Los Angeles, CA 90033, USA

## Abstract

Orb2 is a functional amyloid that plays a key role in *Drosophila* long-term memory formation. Orb2 has two isoforms that differ in their N-termini. The N-terminus of the A isoform (Orb2A) that precedes its Q-rich prion-like domain has been shown to be important for Orb2 aggregation and long-term memory. However, besides the fact that it forms fibrillar aggregates, structural information of Orb2 is largely absent. To understand the importance of the N-terminus of Orb2A and its relation to the fibril core, we recorded solid-state NMR and EPR data on fibrils formed by the first 88 residues of Orb2A (Orb2A88). These data show that the N-terminus of Orb2A not only promotes the formation of fibrils, but also forms the fibril core of Orb2A88. This fibril core has an in-register parallel β-sheet structure and does not include the Q-rich, prion-like domain of Orb2. The Q-rich domain is part of the unstructured region, which becomes increasingly dynamic towards the C-terminus.

Long-term memory (LTM) is the result of a change in efficiency and number of synaptic connections, which requires a change in local protein composition at the level of the synapse. Cytoplasmic polyadenylation element binding proteins (CPEBs) are known regulators of protein expression at the synapse. CPEBs bind dormant mRNA and regulate their polyadenylation and thereby translation^1^. Some CPEBs become activated and initiate mRNA polyadenylation by forming amyloid-like aggregates. This was first described in *Aplysia californica* where the aggregated form of the neuronal isoform of CPEB is required for long-term facilitation^2^,^3^. A similar mechanism was found for CPEB3^4^ in mice and the CPEB protein Orb2 in *Drosophila melanogaster*. Orb2 is a key regulator of LTM^5^ and is enriched in the synaptic region of neurons, where it can remain soluble or form amyloid-like aggregates. The ability of Orb2 to aggregate *in vivo* correlates with the ability of flies to form long-term memories^6^. The aggregated form of Orb2 functions as a translation activator whereas the soluble form actively represses translation of target mRNA^7^. Blocking amyloid formation using small anti-amyloidogenic peptides also blocks LTM^8^. These findings strongly suggest that Orb2A amyloid formation is an important signaling event in LTM, thereby placing Orb2A among an increasing number of amyloids that are found in non-disease related, functional contexts^9^.

There are two Orb2 isoforms, Orb2A and Orb2B. Both isoforms share a C-terminus containing domains common to all CPEBs: two RNA recognition motifs (RRMs) and a zinc finger^10^. Both isoforms have a glutamine-rich (Q-rich) domain, which is reminiscent of the elongated polyglutamine (polyQ) domains responsible for several neurodegenerative disorders, such as Huntington’s Disease. This domain is also similar to the asparagine and glutamine rich domains found in yeast prions. In fact the N-terminus of Orb2A can functionally replace the prion domain of the yeast prion Sup35 in yeast^8^. Orb2A and Orb2B differ at their N-termini. Orb2A has only 21 residues preceding the Q-rich domain with residues 1–9 unique to Orb2A. In contrast, the N-terminus of Orb2B is a 178 residue long, serine-rich domain preceding the Q-rich domain (see Fig. 1A).

Recombinant Orb2A, Orb2B, and corresponding N-terminal fragments form amyloid fibrils *in vitro*. During aggregation A11 positive aggregates are formed initially, while later stages and the mature fibrils are recognized by the OC antibody, similar to pathological amyloids^8^. *In vivo* however, Orb2A, and in particular its Q-rich and N-terminal domains, is essential for the formation of amyloid-like aggregates by the isoform Ob2B and for LTM. Orb2A is very rare and amyloid-like Orb2 aggregates extracted from *Drosophila* heads are primarily composed of Orb2B^6^. Majumdar and co-workers showed that the first 88 residues of Orb2A (Orb2A88) were able to form amyloid-like aggregates in S2 cells. N-terminal deletions of Orb2A or even an F5Y point mutation and many other mutations at that residue significantly reduced Orb2A’s ability to aggregate and promote LTM *in vivo*^6^,^8^. Furthermore, Orb2A functions without its RNA binding domain (RBD), whereas Orb2B is still able to induce LTM in the absence of its Q-rich domain^11^. Taken together, these data suggest that the N-terminus of Orb2A is essential for Orb2B fibril formation and function. However, besides electron micrographs showing that Orb2 forms fibrillar aggregates, no structural information of Orb2 fibrils is known. In particular, the location of Orb2’s fibril core, the structural importance of the Q-rich and N-terminal domains, whether or not these fibrils are β-sheet rich amyloid, and if these β-sheets are in an in-register parallel β conformation, is not known.

In the following report, we present the first detailed structural study of fibrils formed by Orb2A88 to address two specific questions: (1) What is the role of the N-terminal domain of Orb2A in amyloid formation? (2) What is the exact location of the amyloid core of Orb2A88? We find that the N-terminal domain of Orb2A can form amyloid by itself and that the Q-rich domain is less important for amyloid formation than originally thought. These results give a direct structural explanation for why Orb2A can aggregate more readily than Orb2B.

## Results

### Orb2A has an aggregation-prone N-terminal sequence

**Figure 1:**
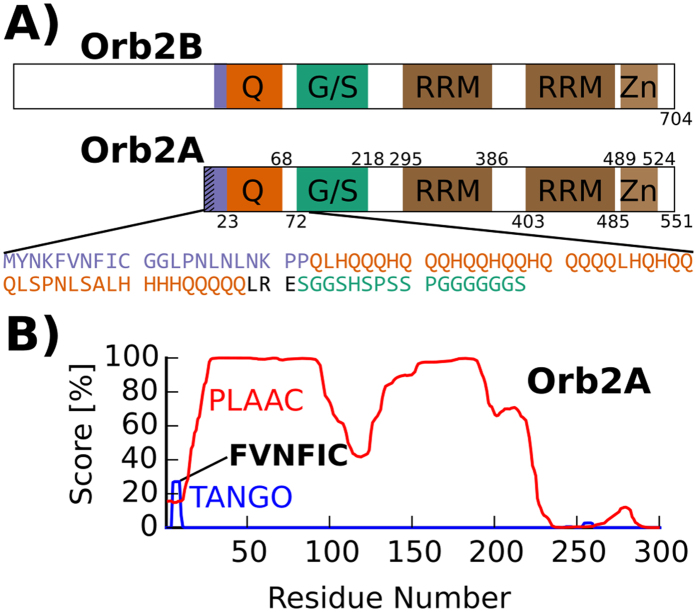
Sequence analysis of Orb2 shows aggregation propensity of N-terminal domain. (**A**) Domain structure of Orb2B and Orb2A. The glutamine/histidine rich domain (Q), glycine and serine rich domain (G/S), the two RNA recognition motifs (RRM) and the C-terminal zinc finger (Zn) are highlighted. In the addition the sequence of the first 88 amino acids of Orb2A is given. (**B**) Sequence analysis of Orb2A using the programs TANGO (blue trace) and PLAAC (red trace) predicts a significant aggregation propensity for the N-terminal residues specific to Orb2A (TANGO) and the existence of a low-complexity prion-like domain (PLAAC). The output score of both programs in % is plotted against the N-terminal sequence of Orb2A.

To identify the amyloid forming domain of Orb2A, we analyzed its sequence using the programs TANGO^12^ and PLAAC^13^. TANGO detects short amino acid sequences that have a high aggregation propensity based on the physicochemical principles underlying β-sheet formation. On the other hand, PLAAC detects domains with prion-like amino acid composition, especially Q and N rich domains that are often found in yeast prions. As can be seen from the blue trace in Fig. 1B, TANGO detected the N-terminal sequence FVNFIC (i.e. Orb2A 5–10) to have significant aggregation propensity. Interestingly, this short peptide includes the F5 residue which was shown to be important for the aggregation of Orb2A and LTM in *Drosophila*^6^. TANGO detected additional sequences with high aggregation propensity inside the C-terminal RBD that are not specific to Orb2A and were also detected in other CPEBs (data not shown). As can be seen from the red trace in Fig. 1B, PLAAC not only identified the Q-rich domain of Orb2A to be prion-like but also the glycine, serine-rich (G, S-rich) domain succeeding the Q-rich domain.

### The N-terminal domain of Orb2A is sufficient for fibril formation

**Figure 2:**
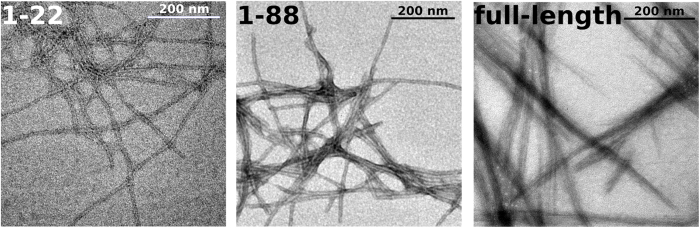
The N-terminus of Orb2A forms amyloid fibrils without the glutamine-rich domain. Negative stained electron micrographs of fibrils formed by Orb2A22, Orb2A88, and full-length Orb2A.

Which of the regions identified by TANGO and PLAAC are necessary for amyloid formation? To answer this question and narrow down the N-terminal location of Orb2A’s fibril core, we tested the following Orb2A constructs for their ability to form amyloid fibrils: (i) the N-terminal domain of Orb2A [i.e. the first 22 residues of Orb2A (Orb2A22)], which include the sequence detected by TANGO; (ii) the first 88 residues of Ob2A (Orb2A88) which include the Q-rich and part of the G/S-rich domain^8^ and were shown to form puncta in cell culture^6^; and (iii) full-length Orb2A. Fibril formation was initiated by bringing the protein constructs from denaturing to renaturing conditions (see Methods). As can be seen in the electron micrographs in Fig. 2, all protein constructs formed fibrillar aggregates under the same conditions. These data show that the N-terminus of Orb2A alone is sufficient to form fibrils *in vitro*. The morphology of these fibrils is different probably because Orb2A88 and in particular full-length Orb2A have additional domains that do not contribute to the fibril core but change their appearance and ability to form bundles.

### The static β-sheet core is located within the N-terminal domain of Orb2A88

**Figure 3:**
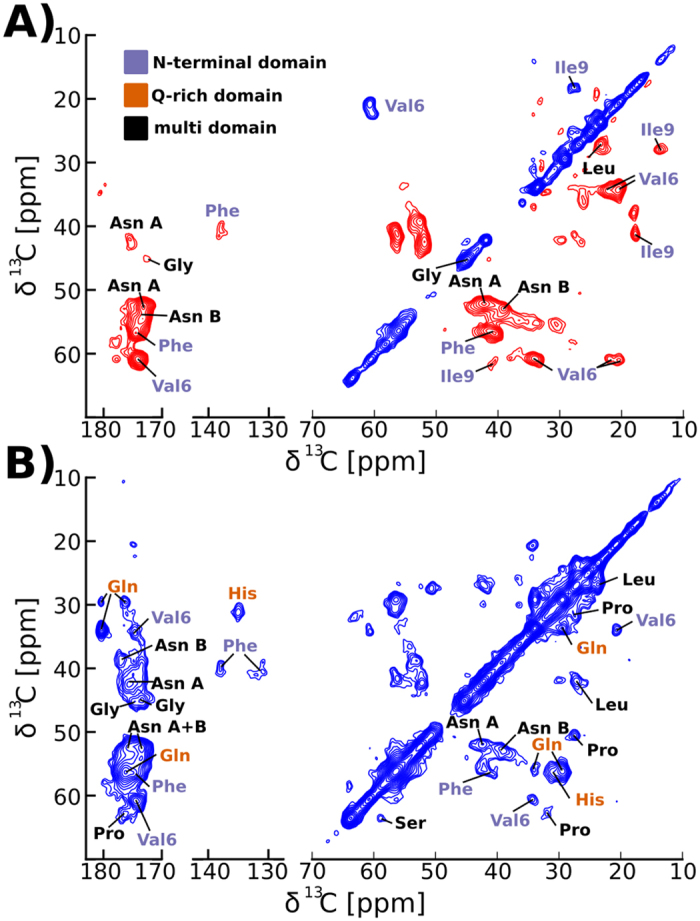
The static domain of Orb2A88 is dominated by residues found in the first 22 residues. (**A**) The strongest cross peaks of the DREAM spectrum of Orb2A88 recorded at 25 kHz MAS and 0 °C belong to residues that are exclusive to or prevalent in the N-terminal 22 residues of Orb2A. Positive and negative signal is shown with blue and red contours, respectively. (**B**) The 2D DARR spectrum recorded under the same conditions shows additional cross peaks of residues such as Gln and His that are not found in the N-terminus. Only positive contours are shown.

Do the N-terminal residues preceding the Q-rich domain form the core of Orb2A88 fibrils alone, or does the Q-rich domain, if present, also form part of the amyloid core of Orb2A88? To answer this question, we recorded solid-state NMR spectra of uniformly ^13^C-^15^N labeled Orb2A88, which includes both the N-terminal and the Q-rich domains. Many amyloid fibrils show a considerable amount of structural and dynamical heterogeneity^14^,^15^. Using dipolar coupling based solid-state NMR methods such as CP and DREAM recoupling, we recorded NMR spectra that only show the most static domains of the protein. These static domains often coincide with the core of the amyloid fibril^14^,^16^,^17^. As can be seen from Fig. 3 the spectral quality with ^13^C linewidths between 0.5 and 1 ppm is comparable to what has been observed for other amyloid fibrils as e.g. formed by the Aβ peptide^18^. We found that Orb2A88 forms fibrils under a variety of different conditions (pH 5–9, 100–200 mM NaCl or KCl, 0–1 M urea, 0–1 mM CaCl_2_, 0–1 mM MgCl_2_, 0–10% glycerol, 4 °C–25 °C) and our NMR spectra of Orb2A88 fibrils showed no batch dependence nor did they depend on whether they were prepared quiescent at 4 °C or agitated at 25 °C, in the presence and absence of glycerol, MgCl_2_, and 100–200 mM NaCl.

We identified the amino acid types in the 2D spectra of Fig. 3 by identifying collections of resonance frequencies belonging to the same amino acid and comparing them to average ^13^C resonance frequencies^19^. Site-specific assignments can be determined for amino acids that are only found once in the sequence of Orb2A88. The most intense cross peaks we detected in our dipolar based ^13^C-^13^C DREAM 2D correlation spectra recorded at 0 °C come from amino acids that are exclusive to the N-terminal domain of Orb2A88 (see Fig. 3A). In particular, we assigned the most intense cross peaks in the DREAM spectra to the only Val (V6) and Ile (I9) residues in Orb2A88. Furthermore, we detected very strong Phe and Asn signals. Phe is exclusive to the N-terminus (F5 and F8), and of the 6 Asn in Orb2A88 only one (N55) is found outside the N-terminal domain. The 2D DREAM spectra also contained weaker signals belonging to Gly and Leu side chains. Interestingly, the DREAM spectra did not show Gln cross peaks. In contrast, we detected strong Gln signals in the ^13^C-^13^C 2D DARR spectrum shown in Fig. 3B. The 2D DARR experiment performs polarization transfer via a different mechanism than the DREAM experiment^20^ and shows cross peaks from additional amino acid types. Besides Gln, we were able to detect resonances belonging to His, Pro, and Ser in our corresponding 2D DARR spectrum (Fig. 3B). Many of these amino acids, namely Gln, His, and Ser, are not found in the N-terminal domain of Orb2A88. We repeated these DREAM and DARR spectra at 25 °C and although the cross peak intensity was reduced, these spectra showed the same set of amino acid residues (see Supplementary Fig. S1).

Comparing the chemical shifts of the amino acids identified in the DREAM spectrum with tabulated chemical shift values for the corresponding residues in β-sheet, α-helical, and random coil conformations^21^ showed that all residues in this spectrum were compatible with a β-sheet conformation, except for Asn B which is between a random coil and β-sheet chemical shift. The additional amino acids detected in the DARR (e.g. Gly, Pro, Gln, His, and Ser) had chemical shifts compatible with a random coil environment (see Supplementary Table S1).

### Dynamic domains of Orb2A88 are located at the C-terminus

**Figure 4:**
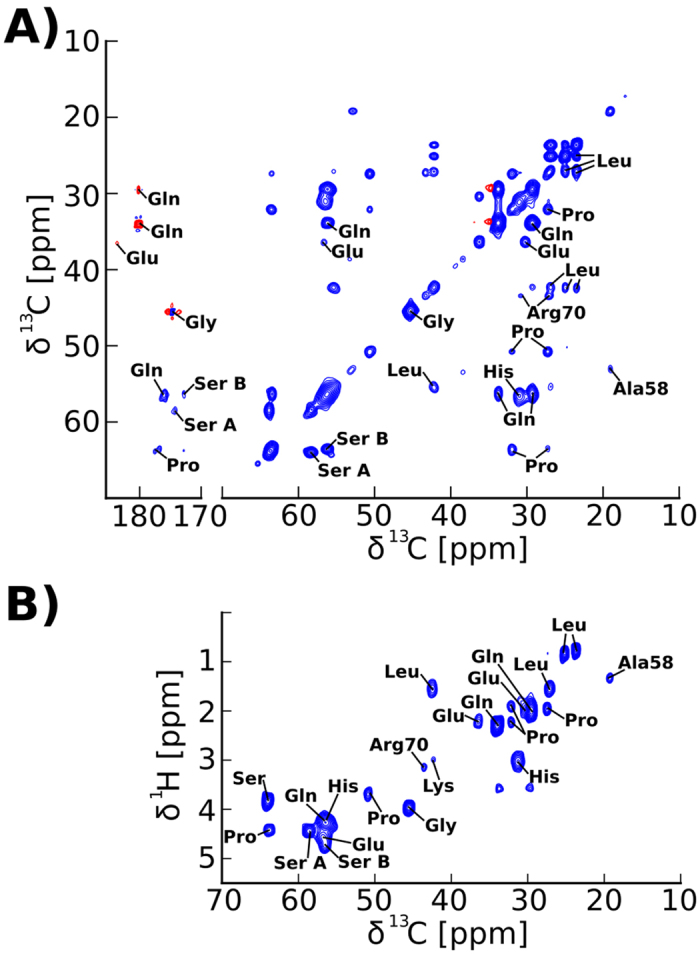
Dynamic domains of Orb2A88 are located in the Q-rich domain and the G/S-rich C-terminus. (**A**) 2D ^13^C-^13^C INEPT adiabatic TOBSY spectrum of Orb2A88 recorded at 25 °C and 24.252 kHz MAS. (**B**) 2D ^1^H-^13^C INEPT HETCOR spectrum recorded at 25 kHz MAS, 25 °C. Amino acid type assignments are shown in both spectra. None of the amino acids that are exclusive to the first 22 amino acids of Orb2A could be detected in these spectra and the strongest signals come from residues that are prevalent in the C-terminus of Orb2A88.

The most dynamic domains of our Orb2A88 fibril preparations were detected using an initial ^1^H–^13^C refocused INEPT that only works in the presence of relatively small ^1^H R_2_^*^ relaxation rates. In fully protonated systems and at the MAS frequencies we used, small ^1^H R_2_^*^ rates indicate drastically reduced dipolar couplings due to motional averaging. Due to the absence of dipolar couplings, we used the through bond, J-coupling based adiabatic TOBSY NMR pulse sequence for ^13^C-^13^C correlation spectra. The dynamic domains are dominated by amino acid residues that are predominantly found in the Q-rich domain and the C-terminus of Orb2A88, as can be seen from the ^13^C-^13^C adiabatic TOBSY and ^1^H-^13^C INEPT HETCOR spectra recorded at 25 °C shown in Fig. 4A and B, respectively. We detected signals from the only Ala (A58) and Arg (R70) in these spectra and signals from Ser, Gln and Glu amino acids that are absent from Orb2A88’s N-terminal domain. Furthermore, we identified resonances from Leu, Pro, and Gly, residues which are found throughout Orb2A88. We repeated the adiabatic TOBSY and INEPT HETCOR spectra at 0 °C, and although the cross peak intensity was reduced they showed the same set of amino acid residues (see Supplementary Fig. S2). The comparison of the chemical shifts of these residues with tabulated values^21^ shows that they are all compatible with a random coil conformation (see Supplementary Table S2). This result is further supported by the pronounced dynamics of these residues. That these dynamic residues originate from the amyloid fibrils and not from soluble Orb2A88 is supported by i) the intensity of the adiabatic TOBSY spectra, which is comparable to the 2D ^13^C-^13^C spectra of the static domains shown in Fig. 3, ii) the fact that the fibrils were washed extensively before packing the NMR samples (see Methods), iii) the absence of any signals from residues unique to the N-terminus of Orb2A88, and iv) that the intensity of the spectra strongly depends on the temperature of the sample, indicating that they do not originate from protein tumbling in solution but from domains outside the fibril core whose dynamic increases with temperature.

### Orb2A88 N-terminal residues form an in-register parallel β-sheet

**Figure 5:**
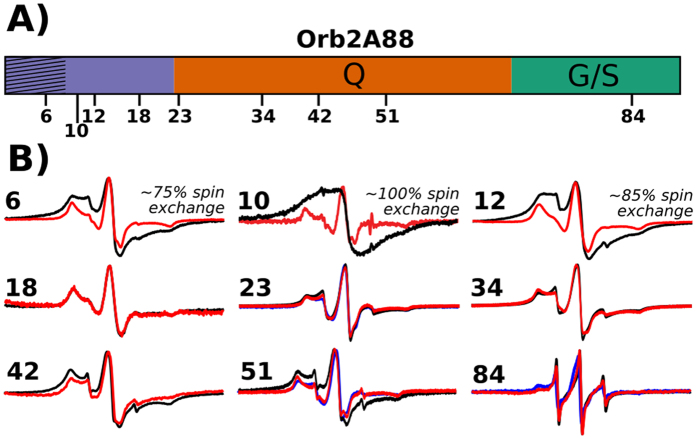
EPR spectra show that the N-terminal amyloid core of Orb2A88 is in an in-register parallel β-sheet conformation. (**A**) Domain structure of Orb2A88 showing positions that were spin labeled. (**B**) EPR spectra of Orb2A88 fibrils spin labeled at the indicated positions. 100% MTSL labeled spectra are shown in black. 10% MTSL labeled samples that were diluted with an MTSL analogue are shown in red. As an additional control we made 10% MTSL labeled spectra of residue 23, 51, and 84 that were diluted with the Cys free C10M mutant instead (shown in blue). Spectra are shown at same amplitude. The percentage of spin exchange calculated from comparing 10% with 100% labeled spectra is given when present. The single EPR line seen for the 100% labeled sample in contrast to the 10% labeled samples is indicative of spin exchange often observed in parallel, in-register β-sheet amyloids^22^.

Does the static amyloid core of Orb2A88 form an in-register parallel β-sheet structure similar to many other amyloid fibrils^22^ or does it have a different intermolecular geometry as e.g. seen in fibrils formed by huntingtin exon-1 (HTT_ex1_)^23^? To answer this question, we recorded EPR spectra of Orb2A88 samples that were MTSL labeled at various sites throughout the sequence as illustrated in Fig. 5A. We took electron micrographs of selected samples to make sure that the MTSL label did not interfere with the fibril formation of Orb2A88.

The comparison between EPR spectra of 10% and 100% spin labeled samples reveals line broadening at several sites due to intermolecular spin-spin interactions (see Fig. 5B). E.g. for residue 10, the already broad EPR spectrum of the 10% labeled sample becomes a single, broad EPR line in the 100% labeled sample. Single EPR lines for MTSL labels are the result of spin exchange that occurs only when multiple spin labels come into close contact (<6 Å). This clustering of spin labels is typically observed in in-register parallel β-sheets where the side chains of the same residue in each monomer stack on top of each other^22^. Besides residue 10, significant amounts of spectral components indicative of spin exchange were also present in the EPR spectra of residues 6 (~75% exchange) and 12 (~85% exchange). Overall this amount of spin exchange is similar to what was previously observed in IAPP fibrils which are also known to be in-register parallel^24^. An out-of-register parallel β-sheet structure would not produce any spin exchange since the spin labels would be spaced by more than 6 Å. The same is true for out-of-register antiparallel β-sheets. An in-register, antiparallel β-sheet could not lead to spin exchange spectra except for one residue in the center of the β-strand. The fact that we observe spin exchange for three N-terminal residues of Orb2A88 shows that a parallel in-register β-sheet structure is the predominant structure of this region^22^. The incomplete spin exchange of residues 6 and 12 could be the result of either incomplete spin labeling or structural heterogeneity. We did not observe any spin exchange after residue 12. The small difference between the EPR spectra with 10% and 100% spin label residues 42 and 51 might be the result of a dipolar spin–spin interactions showing that these residues are within 20 Å from each other^25^.

**Figure 6:**
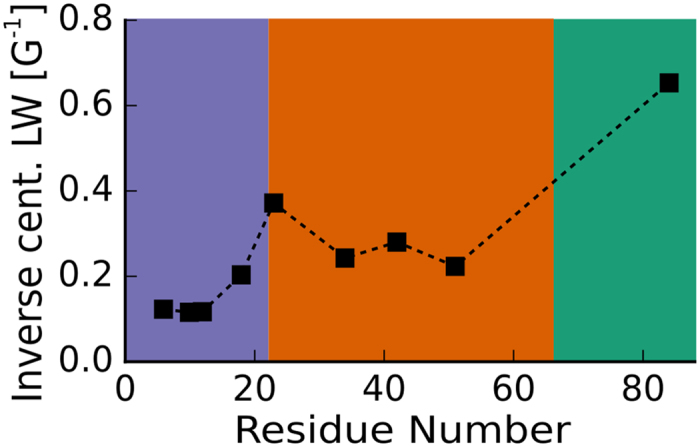
EPR linewidths show that the N-terminal amyloid core of Orb2A88 is static whereas the rest of the protein becomes increasingly dynamic towards the C-terminus. Inverse central linewidths of 10% spin labeled samples. The smaller the inverse EPR linewidth, the less dynamic the corresponding spin label. The data confirm that the most static domain of Orb2A88 is within the N-terminal region and that the C-terminus is highly dynamic.

The inverse central linewidth of EPR spectra recorded on 10% spin labeled samples (i.e. labeled to 10% with MTSL and 90% with an EPR inactive MTSL analogue or the label-free variant Orb2A88 C10M) is not influenced by spin-spin coupling. It increases with increasing dynamics, and is, therefore, a sensitive indicator of dynamics^26^,^27^. As seen from Fig. 6, the inverse central linewidth of EPR spectra from labels in the N-terminal 22 residues is smaller or equal to 0.2 G^−1^, values typical for residues found inside a protein core^26^,^28^. Following the N-terminus, the inverse central linewidth increases towards the C-terminus until it reaches 0.65 G^−1^, a value compatible with a location in flexible loops.

## Discussion

The N-terminal residues specific to Orb2A are important for Orb2 puncta formation in cell culture and LTM in *Drosophila*, and a single point mutation F5Y can diminish puncta formation and LTM^6^. The present study gives a structural explanation for this importance by showing that the N-terminal domain of Orb2A is able to form amyloid fibrils on its own and forms the in-register parallel β-sheet core of Orb2A88 fibrils. In particular, our solid-state NMR data indicate that Phe is part of this in-register parallel β-sheet amyloid core. We were not able to produce Orb2A88 F5Y fibrils in quantities that allowed a direct comparison with our WT Orb2A88 data, which further confirmed the decreased ability of this mutant to form amyloid. Previous studies estimated the amyloid forming propensity of Phe to be higher than that of Tyr^29^, and Phe-Phe pairs are favored in protein cores and in distances compatible with an in-register parallel β-sheet arrangement^30^,^31^. Together, these findings give a possible explanation for the effects of the F5Y mutation.

Although the program PLAAC found the Q-rich domain of Orb2 to be prion-like and despite the similarities of this domain to other Q/N-rich yeast prions and aggregation prone polyQ domains, our combined solid-state NMR and EPR data show that this domain is not part of the in-register parallel β-sheet amyloid core formed by Orb2A88. This initially surprising result can be explained by the presence of many non-Gln residues, especially His, in this domain, which with 43 residues is just above the threshold found for polyQ domains in neurodegenerative diseases^32^. Histidine insertions, in particular, were shown to lower the aggregation propensity of polyQ domains^33^,^34^, which may explain the absence of any β-sheet Gln residues in our spectra. In fact, the Q-rich domain is more dynamic than the amyloid core and in a disordered state as concluded by our chemical shift analysis. On the other hand, His and Gln residues were detected in the DARR spectra, the Q-rich domain gave EPR spectra of intermediate linewidth, and even showed some dipolar interaction by EPR, indicating that this region has some residual structure.

**Figure 7:**
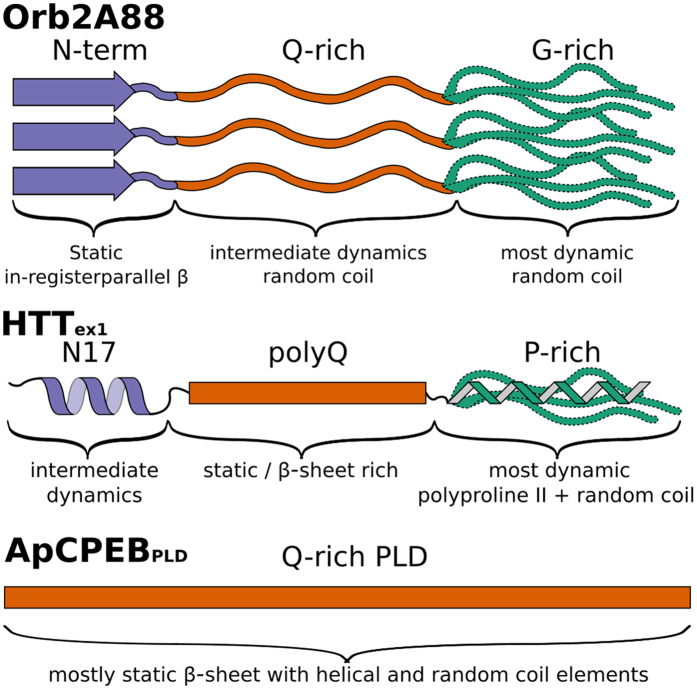
Schematic of the structure and dynamics of Orb2A88 fibrils and comparison with HTT_ex1_ and the prion-like domain of ApCPEB. In Orb2A88, the N-terminal residues preceding the Q-rich domain are the most static part of the fibril and are in an in-register parallel β-sheet conformation (purple arrow). The Q-rich domain (orange) is disordered and less static than the N-terminus. The G/S-rich domain (green) is highly dynamic and in a random coil conformation. The amyloid core of HTT_ex1_, in contrast, is formed by the polyQ domain, whereas the N-terminus and C-terminus are not part of the amyloid core but form a helix with intermediate dynamics and a dynamic mixed polyproline II, random coil conformation, respectively^38^,^39^,^49^. The relatively large Q-rich, prion-like domain (PLD) of ApCPEB is predominantly in a β-sheet conformation and forms the amyloid core of this protein.

The Gly and Ser rich C-terminus of Orb2A88 is the most dynamic part of the fibril and in a random coil conformation. Generally, Gly and Ser rich domains have a high propensity to be intrinsically disordered^35^. We illustrate the structural and dynamic heterogeneity of our Orb2A88 fibrils in the model shown in Fig. 7. In this model the N-terminal domain forms a static in-register parallel β-sheet that is the amyloid core, the Q-rich domain is more dynamic and in an unstructured, random coil conformation, and the C-terminal, G/S-rich domain is most dynamic and disordered.

In contrast to the Q-rich domain of Orb2A88, the previous study on *Aplysia* CPEB (ApCPEB) showed that the static amyloid core of ApCPEB was formed by the Q-rich domain which was predominantly in a β-sheet conformation (with some of the Gln in helical and random coil environments)^36^. ApCPEB’s Q-rich domain is, with 92 residues, significantly longer than the thresholds for polyQ domains in neurodegenerative diseases and contains much longer uninterrupted polyQ stretches than does the Q-rich domain of Orb2, giving a possible explanation for the difference in aggregation behavior (Fig. 7).

The amyloid core formed by HTT_ex1_ with an expanded polyQ domain has a different structure than Orb2A88 and ApCPEB amyloid. Its static amyloid core is formed by the polyQ domain, which is composed of two conformationally distinct forms of Gln that occur at a 1:1 ratio^37^,^38^,^39^. The polyQ domain is β-sheet rich but is not in an in-register parallel β-sheet structure^23^. The 17 residues preceding the polyQ domain of HTT_ex1_ (N17) are not part of the β-sheet amyloid core but were shown to promote the aggregation of HTT_ex1_ into amyloid fibrils by oligomerization or membrane interaction^40^,^41^. N17 retains its helical structure after HTT_ex1_ aggregates into fibrils (Fig. 7)^38^.

Although all three Q-rich proteins, Orb2, ApCPEB, and HTT_ex1_, form amyloid fibrils, we found that they have very different fibril structures and that the Q-rich domain does not contribute to the core in the case of our Orb2A88 fibrils. But what is the function of the Q-rich domain in Orb2? One possibility is that the function of this domain is similar to the native function of polyQ domains found e.g. in HTT where it has been hypothesized to be important for non-amyloidogenic protein-protein interaction^42^. Another possibility is that additional factors might be necessary for this part of the protein to adopt an amyloid structure.

What does this model mean for the structure of full-length Orb2A fibrils and mixed Orb2A, Orb2B aggregates found *in vivo*? Orb2A’s N-terminal domain not only promotes aggregation, similar to the N17 domain of HTT, but forms the core of Orb2A88 fibrils itself. This helps explain why this domain is essential for Orb2A aggregation. However, full-length Orb2A aggregates more completely in cell culture compared to Orb2A88^6^ and longer Orb2A fragments are needed to obtain translation activation comparable to full-length Orb2A^7^. Furthermore, Orb2B lacks some of the key N-terminal residues we identified to form the amyloid core of Orb2A88 fibrils and only a few residues of this core are common in both Orb2A and Orb2B. It is unlikely that these residues are sufficient for stabilizing Orb2A-Orb2B co-aggregates. The existence of several distinct domains that are important for seed formation and aggregate stability was recently reported for aggregates of mammalian CPEB3^4^. Therefore, it is possible that additional domains play a role in stabilizing Orb2A-Orb2B co-aggregates. While the N-terminal domain specific to Orb2A is able to form amyloid by itself and is necessary for aggregate nucleation and long-term memory, the other domains of Orb2 might play important roles not only for RNA binding but also for aggregate stabilization. These could include the Q-rich domain that might not be important for Orb2A aggregation itself but the formation of Orb2A-Orb2B co-aggregates. We are currently investigating the structure of these domains with the goal of understanding how Orb2 aggregation regulates the polyadenylation and thereby translation of its target mRNA. These results will ultimately help reveal the molecular mechanism of LTM, potentially aid treatment for memory deficiencies, and contribute to our understanding of amyloid diseases via comparison with disease-related amyloids.

## Methods

### Plasmids and protein constructs

All Orb2A88 and full-length Orb2A constructs were cloned into pET28b expression vectors. For the spin-labeled variants the cysteine at position 10 in the wild type Orb2A88 plasmid was mutated to methionine using site-directed mutagenesis. All additional cysteine mutations, except for Q23C, Q34C and G12C, were introduced into this C10M plasmid also using the QuickChange II XL Site Directed Mutagenesis Kit (Agilent Technologies Inc, Santa Clara, CA). Mutants Q23C, Q34C, and G12C were directly ordered from Genscript USA Inc.

### Protein expression and purification

For Orb2A88 *E. Coli* Rosetta™ 2 (DE3) (EMD Millipore, Billerica MA) cell cultures were grown at 30 °C for 15–18 hours. Cultures were then diluted into LB Miller medium containing appropriate antibiotics and grown at 37 °C until cell density reached an optical density at 600 nm of 0.6. 1 mM Isopropyl 1-thiol-βD-galactopyranoside (IPTG) was then added to induce protein expression and temperature was decreased to 25 °C for 15–18 hours. The G12C, Q23C, and Q34C mutants were expressed in a similar manner, but using BL21 (DE3) cell cultures. ^13^C-^15^N labeled Orb2A88 was expressed in M9 minimal medium following a protocol by Marley and co-workers^43^. Full-length Orb2A was expressed in a similar manner, but at 32 °C for 4–5 hours. Cell pellets were collected using centrifugation at 4,000 rpm for 20 minutes using a Sorvall SLC-6000 rotor (Thermo Fisher Scientific Inc.), and were then immediately used or stored at −80 °C. Cells were resuspended and lysed in Denaturing Buffer (10 mM Tris, pH 8.0, 8 M urea, 100 mM NaH_2_PO_4_ and 0.05% v/v β-mercaptoethanol) and lysis was carried out using Cell Disruptor Sonicator^TM^ (Heat Systems Model W-220F). Soluble parts were then isolated through centrifugation at 20,000 rpm for 20 minutes using a Sorvall ss-34 rotor. The resulting supernatant was poured into a pre-equilibrated Ni-NTA column and incubated on a shaker at room temperature for a minimum of 1 hour. The flowthrough was subsequently collected and the column was washed with Denaturing Buffer containing 0.5% Triton X-100 followed by Denaturing Buffer containing 500 mM NaCl. The column was also washed with Denaturing Buffer pH 6.75 and Renaturing Buffer (200 mM NaCl, pH 8.0, 50 mM NaH_2_PO_4_, 10% glycerol, 0.05% v/v β-mercaptoethanol) with 20 mM imidazole. Protein was eluted with an imidazole step gradient in Renaturing Buffer. The majority of protein eluted in the 100 mM and 150 mM imidazole fractions. Resulting protein was either used immediately or frozen as 2 mL aliquots using liquid N_2_ and stored at −80 °C.

For Orb2A, cells were resuspended in Extraction Buffer (50 mM Tris, 100 mM NaCl, 0.5% v/v Triton X-100, 0.05% v/v β-mercaptoethanol, 1 mg/ml lysozyme and 1X Pierce Protease Inhibitor). Following sonication, insoluble parts were isolated via centrifugation at 10,000 rpm for 15 minutes. The insoluble fraction was resuspended in Extraction Buffer and the process was repeated. The insoluble fraction of this procedure was then resuspended in pH 8.0 Full Length Denaturing Buffer (FL-Denaturing Buffer) (6 M guanidine hydrochloride, 250 mM NaCl, 100 mM Na_2_HPO_4_ and 10% v/v glycerol) and sonicated again. The mixture was then left on a shaker at room temperature for at least overnight. The soluble fraction was then isolated by centrifugation at 20,000 rpm for 20 minutes. Orb2A was also purified using a Ni-NTA column, the column was washed with Fl-Denaturing Buffer pH 6.7, and eluted with FL-Denaturing Buffer pH 3.75.

### Spin labeling

Spin labeling for EPR studies was done using a protocol similar to the one described by Bugg and co-workers^23^. Orb2A88 aliquots were desalted using a PD-10 desalting columns (GE Healthcare, Buckinghamshire, UK) into Renaturing Buffer, pH 7.4. Eluted samples were collected on ice and concentrations were measured using UV absorbance at 280 nm (ε = 1490 M^−1^ cm^−1^, pathlength = 1 cm). Spin labeling was subsequently achieved through incubation with 5.5 molar excess of MTSL (1-Oxyl-2,2,5,5-tetramethyl-∆3-pyrroline-3-methyl Methanethiosulfonate) spin label (Toronto Research Chemicals, Inc., North York, ON, Canada) overnight at 4 °C. 10% labeled samples were labeled in a similar manner, but using either a combination of MTSL spin label and MTSL analog (1-Acetyl-2,25,5-tetramethyl-3pyrroline-3-methyl Methanethiosulfate) or by diluting the MTSL spin labeled sample with the Cys free C10M mutant. Excess label was removed through dialysis against Renaturing Buffer (200 mM NaCl, pH 7.4, 50 mM NaH_2_PO_4_, 10% glycerol).

### Fibril formation

For NMR analysis, recombinant Orb2A88 was dialyzed 200 mM NaCl, pH 7.4, 50 mM NaH_2_PO_4_, 10% glycerol, 1 mM DTT and incubated at room temperature for up to 2 weeks to form amyloid fibrils. For Electron Microscopy analysis, recombinant Orb2A and Orb2A88 samples were exchanged into 10 mM HEPES, pH 7.6, 100 mM KCl, 1 M Urea, and 1 mM DTT using dialysis and a PD-10 desalting column, respectively. Samples were then incubated on a shaker at room temperature for up to 2 weeks. To form fibrils of Orb2A22, lyophilized powder of this peptide (Anaspec, Fremont, CA) was redissolved in in the same buffer as Orb2A and Orb2A88, at a concentration of 45 μM, and was incubated under the same conditions.

### Sequence analysis

The sequence of Orb2A was analyzed using the programs TANGO^12^ and PLAAC^13^ using standard parameters to detect regions of high aggregation propensity and domains of prion-like amino acid composition, respectively.

### Solid-state NMR spectroscopy

All spectra were recorded on an Agilent DD2 600 MHz solid-state NMR spectrometer (Agilent Technologies Inc, Santa Clara, CA). Fibril samples were washed repeatedly with deionized water, sedimented by centrifugation (13,500 rpm; Eppendorf FA45-30-11 rotor; 20 min) and packed into 1.6 mm magic angle spinning (MAS) rotors. All spectra were recorded using a T3 1.6 mm probe operating at 25 kHz MAS if not mentioned otherwise. 200 kHz and 100 kHz hard pulses were applied on ^1^H and ^13^C, respectively. The recycle delay was 3 s for all spectra. ^1^H-^13^C cross polarizations (CPs) were done using a Hartman-Hahn match of 60 kHz on ^13^C and 85 kHz on ^1^H with a 10% amplitude ramp. 120–160 kHz TPPM (two pulse phase modulation) ^1^H decoupling was used during direct and indirect detection.

^13^C-^13^C DARR (dipolar assisted rotational resonance) spectra^44^ were recorded using a 25 kHz ^1^H recoupling field during the mixing period of 50 ms, a spectral width of 50 kHz in both dimensions, and 32 and acquisitions were recorded for each of the 400 complex t_1_ increments. ^13^C-^13^C DREAM (dipolar recoupling enhanced by amplitude modulation)^45^ spectra were recorded using a mixing time of 4.5 ms and a tangential amplitude modulation (Δ/2π = 3 kHz and β/2π = 1.4 kHz^46^ around the HORROR [homonuclear rotational resonance] condition of 12.5 kHz). The spectral width was 50 kHz in both dimension and 48 to 64 acquisitions were co-added for each of the 400 to 600 t_1_ increments. ^13^C-^13^C adiabatic TOBSY (total through bond spectroscopy) spectra^47^ were recorded as described previously^14^ using an initial refocused INEPT (insensitive nuclei enhanced by polarization transfer), 7.72 ms of adiabatic TOBSY recoupling (i.e. 8 cycles of 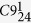 WiW recoupling with 55 μs per WURST-8 pulse of 90 kHz ^13^C peak amplitude), no ^1^H decoupling during the mixing time, and a MAS frequency of 24.242 kHz. The spectral width of the adiabatic TOBSY spectra was 50 kHz in both dimensions and 32 acquisitions were co-added for each of the 600 complex t_1_ increments.

^1^H-^13^C HETCOR (heteronuclear correlation) spectra were recorded using refocused INEPT. The indirect ^1^H dimension had an 180° ^13^C refocusing pulse and a spectral width of 10 kHz. The direct ^13^C dimension had a spectral width of 50 kHz. Sixteen acquisitions were co-added for each of the 200 complex t_1_ increments, and 35 kHz Waltz decoupling was applied during acquisition.

All spectra were referenced to 4,4-dimethyl-4-silapentane-1-sulfonic acid (DSS) using adamantane as external reference^48^.

### EPR Spectroscopy

Fibril formation was confirmed by measuring Thioflavin T fluorescence at 482 nm (FP-6500 spectrofluorometer, Jasco, Inc., Easton, MD). Fibrils were collected using centrifugation at 13,500 rpm for 10 minutes (5840R centrifuge, F45-30-11 rotor, Eppendorf AG Hamburg, Germany). The soluble portion was removed and the remaining pellet was washed repeatedly with deionized water. The sample was then loaded into a boro capillary tube (0.6 mm inner diameter, 0.84 mm outer diameter, Vitro-Com, Mt. Lakes, NJ).

Continuous wave EPR spectra were collected at room temperature using a Bruker X-band EMX Spectrometer (Bruker Biospin Corporation). Each sample was scanned 15 times using scan width of 150 gauss in an HS cavity and microwave power of 12.60 milliwatts. In a some cases (i.e. residues 10, 12, 23, and 34), spectra of the supernatant were subtracted from the fibril spectra to get cleaner fibril spectra.

### Electron Microscopy

Fibrils were adsorbed onto copper mesh electron microscopy grids (Electron Microscopy Sciences, Hatfield, PA) for 5 min. These grids were negatively stained with 1% uranyl acetate for 2 min, rinsed with deionized water and dried. Subsequently, the grids were examined with a JEOL JEM-1400 electron microscope (JEOL, Peabody, MA) at 100 kV and photographed using a Gatan digital camera.

## Additional Information

**How to cite this article**: Cervantes, S. A. *et al*. Identification and Structural Characterization of the N-terminal Amyloid Core of Orb2 isoform A. *Sci. Rep.* **6**, 38265; doi: 10.1038/srep38265 (2016).

**Publisher's note:** Springer Nature remains neutral with regard to jurisdictional claims in published maps and institutional affiliations.

## Supplementary Material

Supplementary Information
