# Supplementary material for: Identification and Structural Characterization of the N-terminal Amyloid Core of Orb2 isoform A
Source: Sci Rep. 2016 Dec 6;6:38265. doi: 10.1038/srep38265 (PMC5138630; doi:10.1038/srep38265)
Supplement: Supplementary Information [file srep38265-s1.pdf]

Supplementary Information for:

# Identification and Structural Characterization of the N-terminal Amyloid Core of Orb2 isoform A

**Silvia A. Cervantes[a], Thalia H. Bajakian[a], Maria A. Soria[a], Alexander S. Falk[a], Rachel J. Service[a,b], Ralf Langen[a], Ansgar B. Siemer[a]**

a) Department of Biochemistry & Molecular Biology and the Zilkha Neurogenetic Institute, Keck School of Medicine of USC, 1501 San Pablo St, Los Angeles, CA 90033, USA

b) Current address: US Army Construction Engineering Research Laboratory, 2902 Newmark Dr, Champaign, IL 61822, USA

**Corresponding author:** Ansgar B. Siemer; 1501 San Pablo St, Los Angeles, CA 90033, USA; Phone: +1 323 442 2720; Email: asiemer@usc.edu

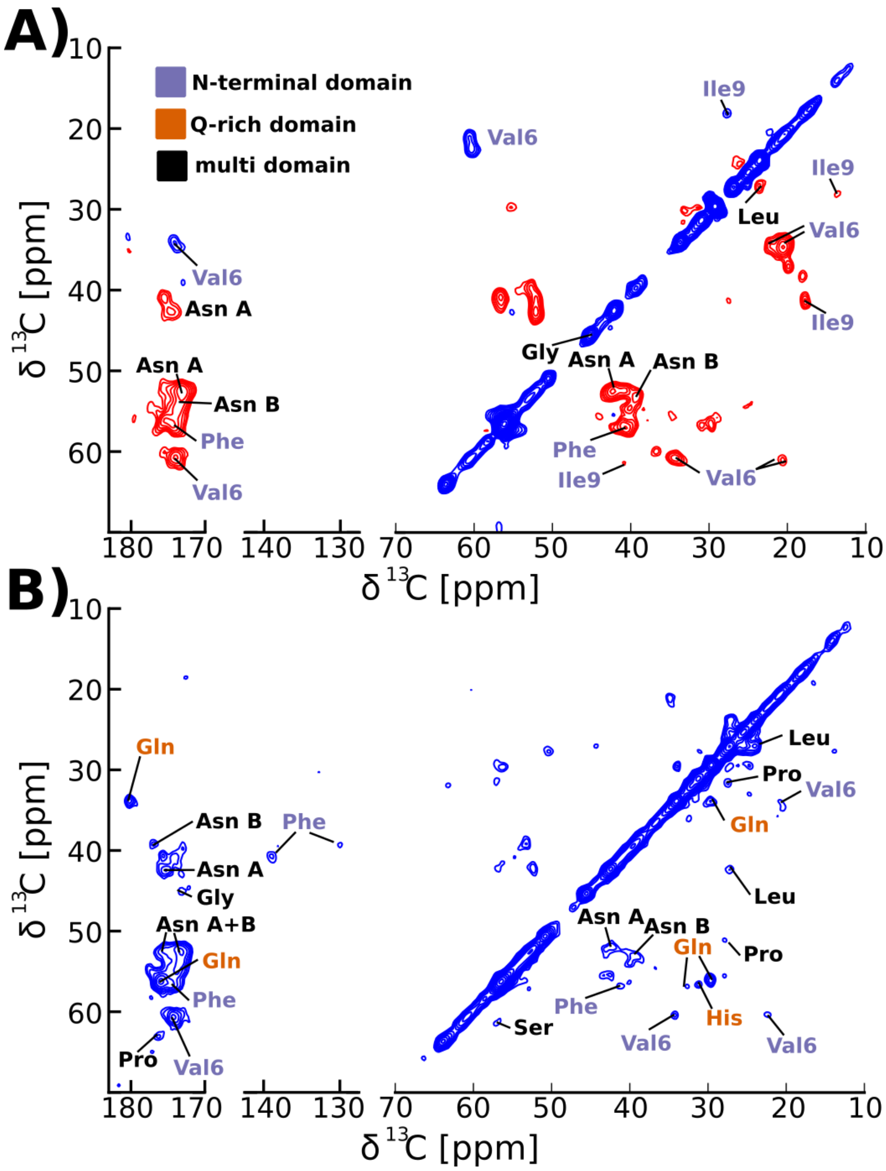


**Figure S1:** Same as Figure 3 but recorded at 25ºC instead of 0ºC. A) 2D $^{13}C$-$^{13}C$ DREAM spectrum of Orb2A88 recorded at 25 kHz MAS and 25ºC. Positive and negative signal is shown with blue and red contours, respectively. B) The 2D DARR spectrum recorded under the same condition. Only positive contours are shown.

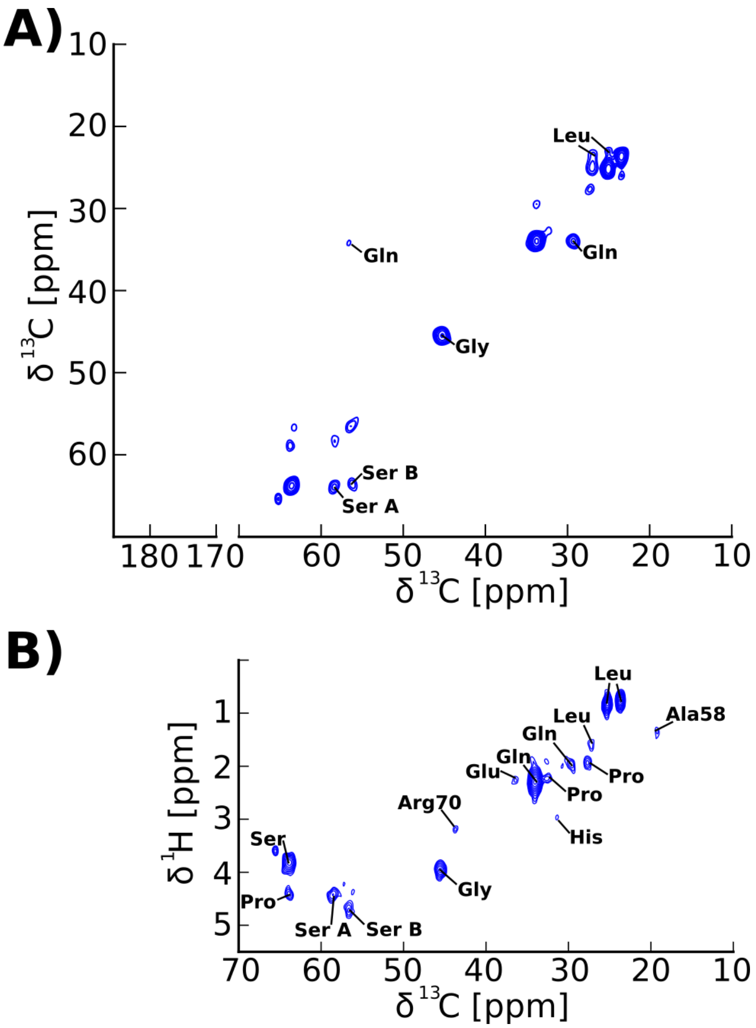


**Figure S2:** Same as Figure 4 but recorded at 0ºC instead of 25ºC. A) 2D $^{13}C$-$^{13}C$ INEPT adiabatic TOBSY spectrum of Orb2A88 recorded at 0ºC and 24.252 kHz MAS. B) 2D $^{1}H$-$^{13}C$ INEPT HETCOR spectrum recorded at 25 kHz MAS, 0ºC. Amino acid type assignments are shown in both spectra. None of the amino acids that are exclusive to the first 22 amino acids of Orb2A could be detected in these spectra and the strongest signals come from residues that are prevalent in the C-terminus of Orb2A88.

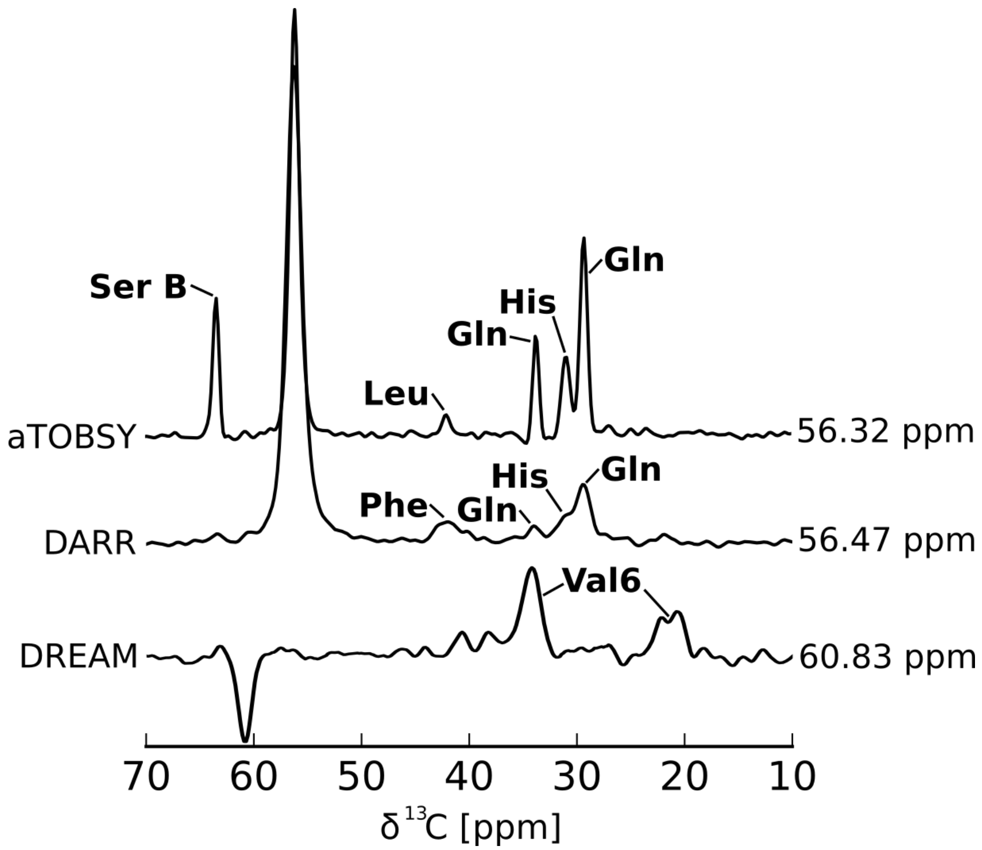


**Figure S3:** 1D slices from the 2D $^{13}C$-$^{13}C$ DREAM, DARR, and aTOBSY spectra of Figures 3A, 3C, and 4B, respectively. $\delta_1$ positions are of each slice and amino acid assignment of the cross peaks are indicated.

**Table S1:** $^{13}$C chemical shifts (in ppm) of the residue assignments shown in Figure 3. The secondary chemical shift for C, CA, and CB calculated using the random coil chemical shifts reported by Wang and Jardetzky [21] is given in parentheses. The $\Delta S = \Delta\delta CA - \Delta\delta CB$ column on the right is the difference in secondary chemical shift between CA and CB. A positive $\Delta S$ value is indicative of an $\alpha$-helical, a negative value of an extended, and values around 0 of a random coil conformation.

| Residue | C | CA | CB | CG | CD | ΔS |
|---|---|---|---|---|---|---|
| Val6 | 174.2 (-1.6) | 60.6 (-1.2) | 34.2 (1.5) | 20.6/22.3 | | -2.7 |
| Ile9 | - | 61.0 (0.4) | 41.1 (2.8) | 27.8/18.0 | 14.1 | -2.4 |
| Phe | 174.5 (-0.7) | 56.4 (-0.5) | 40.9 (1.5) | 138.0 | 131.4 | -2.0 |
| Asn A | 173.3 (-1.7) | 52.0 (-0.9) | 42.3 (4.1) | 175.2 | | -5.0 |
| Asn B | 173.2 (-1.8) | 52.6 (-0.3) | 39.0 (0.8) | 177.0 | | -1.1 |
| Gly | 173.7 (-0.6) | 45.3 (0.0) | | | | 0.0 |
| Pro | 176.1 (-0.81) | 62.9 (-0.6) | 31.8 (-0.1) | 27.3 | 50.8 | -0.5 |
| Gln | 176.0 (0.1) | 56.2 (0.3) | 29.4 (0.7) | 33.8 | 180.1 | -0.4 |
| His | - | 56.9 (1.1) | 30.9 (1.3) | 135.0 | 119.7 | -0.2 |
| Ser | - | 58.4 (0.1) | 63.9 (0.0) | | | 0.1 |

**Table S2:** Chemical shifts (in ppm) of the amino acid assignments shown in Figure 4. The secondary chemical shift for C, CA, and CB given in parentheses and the $\Delta S = \Delta\delta CA - \Delta\delta CB$ column are calculated as described in Table 1.

| Residue | C | CA | CB | CG | CD | HA | HB | HG | HD | ΔS |
|---|---|---|---|---|---|---|---|---|---|---|
| Ala58 | - | 53.0(0.1) | 19.1(0.1) | | | - | 1.3 | | | 0.0 |
| Ser A | 174.2(-0.1) | 58.3(-0.1) | 63.9(0.0) | | | 4.4 | 3.8 | | | -0.1 |
| Ser B | 172.7(-1.6) | 56.3(-2.1) | 63.6(-0.3) | | | 4.7 | 3.8 | | | -1.8 |
| Gln | 176.0 (0.1) | 56.3(0.4) | 29.4(0.7) | 33.8 | 180.2 | 4.3 | 2.0 | 2.3 | | -0.3 |
| His | - | 56.4(0.6) | 31.1(1.5) | 135.1 | 119.9 | 4.3 | 3.0 | | | -0.9 |
| Pro | 176.8(0.1) | 63.5(0.0) | 32.0(0.1) | 27.3 | 50.7 | 4.4 | 1.9/ 2.2 | 2.0 | 3.7 | -0.1 |
| Leu | - | 55.4(0.6) | 42.3(0.4) | 27.0 | 23.5/ 25.0 | - | 1.5 | 1.6 | 0.8/ 0.8 | 0.2 |
| Gly | 174.7(0.4) | 45.3(0.0) | | | | 4.0 | | | | (0.0) |
| Arg70 | - | - | 30.9(0.4) | 27.2 | 43.4 | - | - | - | 3.1 | (-0.4) |
| Glu | | 56.7(0.3) | 30.3(0.3) | 36.3 | 183.7 | 4.5 | 2.0 | 2.2 | | 0.0 |
